# Effect of the vertical facial pattern on the developmental relationship between the nasal bone and maxillary central incisors

**DOI:** 10.1186/s12903-023-02927-x

**Published:** 2023-04-12

**Authors:** Jianwei Shi, Mohammed Sultan Al-Ak’hali, Dingjun Cai, Qiutong Guo, Yuming Cao, Maged S. Alhammadi, Mubarak Ahmed Mashrah, Yang Yang

**Affiliations:** 1 Department of Orthodontics, Affiliated Stomatology Hospital of Guangzhou Medical University, Guangdong Engineering Research Center of Oral Restoration and Reconstruction, Guangzhou Key Laboratory of Basic and Applied Research of Oral Regenerative Medicine, Guangzhou, 510182 Guangdong China; 2 Department of Preventive Dental Sciences, College of Dentistry, Jazan University, Jazan, Saudi Arabia; 3 Department of Periodontology, Faculty of Dentistry, Sana’a University, Sana’a, Yemen; 4 Orthodontics and Dentofacial Orthopedics, Department of Preventive Dental Sciences, Jazan University, Jazan, Saudi Arabia; 5 Department of Implantology, Affiliated Stomatology Hospital of Guangzhou Medical University, Guangdong Engineering Research Center of Oral Restoration and Reconstruction, Guangzhou Key Laboratory of Basic and Applied Research of Oral Regenerative Medicine, Guangzhou, 510182 Guangdong China

**Keywords:** Nasal bone, Maxillary central incisors, Vertical facial patterns, Correlation

## Abstract

**Background:**

This study aimed to investigate the effect of vertical facial patterns on the developmental relationship between the nasal bone and maxillary central incisors.

**Methods:**

In this retrospective comparative study, the lateral cephalograms of 213 subjects (51 Males, 162 Females) with skeletal Class I malocclusion (aged 18–32 years) were classified into three equal groups: (1) hyperdivergent, (2) normodivergent, and (3) hypodivergent facial patterns based on the mandibular plane inclination (S–N/Go-Me). Several sets of measurements were extracted: (1) gradient and length of the nasal bone and maxillary central incisor, (2) the distance from apex and root of the nasal bone, and (3) maxillary central incisor to the true perpendicular from the digitized lateral cephalograms. The significance level was considered at *P* < 0.05.

**Results:**

The inclination angle and length between nasal bone and maxillary central incisor were positively correlated independent of vertical facial type. The inclination angle of the nasal bone in the hypodivergent group was significantly larger than the other two vertical facial patterns. The inclination angle of the maxillary central incisor increased successively in the hyperdivergent, normodivergent, and hypodivergent groups. The length of the nasal bone in the hyperdivergent group was significantly longer than that in the hypodivergent and normodivergent groups. The maxillary central incisor length in the hyperdivergent group was significantly longer than in the hypodivergent group.

**Conclusion:**

A correlation between nasal bone and maxillary central incisors during the growth and development of the maxillofacial region was found. In Class I malocclusion subjects, hypodivergent patients were more likely to have a prominent and relatively short nasal bone and maxillary central incisors and vice versa.

## Background

Other than genetic factors, oro-facial growth and development are affected by several environmental conditions which are related and correlated with each other [1]. As the most prominent organ and central position of the face, the nose has a great impact on facial beauty and craniofacial growth [2]. The nasal shape and height relate to coordinate with other oro-facial structures, including the upper lip, eyes, forehead, and others. For this reason, rhinoplasty is one of the most common and popular operations in plastic surgery forms worldwide [3].

Additionally, maxillary central incisors are located in the center of the mouth, which are the most arresting teeth while talking, smiling, and eating [4]. Therefore, the growth and development of the nose and maxillary central incisors and the relationship between them are worth studying and exploring of this hidden area.

The frontal and maxillofacial regions are developed from neural crest cells in the ectoderm. Migration and differentiation of neural crest cells form the branchial arch and pharyngeal sac during embryonic development, which is a prominent feature of facial development [5, 6]. The branchial arch further develops and comprises many protrusions, including globular, lateral nasal, maxillary, and mandibular processes [6, 7]. The fusion and combination of these protrusions form the maxillofacial region and differentiate into different maxillofacial tissues. For instance, the maxillary incisors, nasal bone, ethmoid bone, vomer, and premaxilla are derived from the globular process [6]. These tissues are developed from the same processes of the same cell, that is, they have the same embryonic origin. Accordingly, they may have the same cell characteristics or biological behaviors [8–10].

Studies have pointed out that the shape and size of the nose are closely related to that of the maxillary incisors, and they suggest that the shape of the nose can predict the width of the maxillary central and lateral incisor [11, 12]. Development of the nasal bone occurs far earlier than that of the permanent maxillary central incisors. Therefore, research addressing the correlation between the nose and the maxillary central incisors might provide powerful evidence to further prove the predictability of maxillary central incisors depending on the development of the nose [13].

Craniofacial development occurs in three-dimensions, including transverse, sagittal, and vertical dimension. Due to the regulation of genes and the external environment, variations appear during craniofacial growth and development, which lead to different facial patterns [14]. In the vertical direction, the craniomaxillofacial complex shows differences in height through the growth of jaw, teeth, and alveolar bone, and the growth and rotation of the mandibular condyle. In several classical measurements and analysis of orthodontics, these height variations are classified into hyperdivergent, normodivergent, and hypodivergent facial groups.

These craniofacial variations are accompanied by dentoalveolar changes; a process that is called dentoalveolar compensation [15, 16].This compensation is capable of maintaining a relatively normal relationship with different facial growth patterns [15]. The dentoalveolar height and the position and inclination of the maxillary incisors can compensate for the vertical and sagittal skeletal discrepancies [17]. Such compensations are intended to remedy craniofacial skeletal disharmony to maintain the overall harmony and proportion of teeth and craniofacial structure.

Maxillary central incisor inclination influences the upper lip [18] and even the aesthetics of the basifacial 1/3 [19, 20] which would provide valuable data to support orthodontic procedures of mixed dentition. Meanwhile, the correlation between nasal bone and maxillary central incisors would also provide reference data for recovering the lost anterior teeth in prosthodontic therapy [21]. However, the etiological relation between the nose and maxillary central incisors, other than morphology, has not been clarified.

Hence, this study was conducted to investigate the effect of vertical facial pattern on the developmental relationship between the nasal bone and maxillary central incisors based on lateral cephalometric measurements.

## Materials and methods

### Study design

This is a retrospective cross-sectional comparative study that was approved by the research ethical committee, Hospital of Stomatology, Guangzhou Medical University, China (No. LCYJ2022053). All study participants or their guardians provided written informed consent to participate in this study. We confirm that all methods were performed in accordance with the relevant guidelines and regulations.

### Study sample size

The sample size was determined utilizing the G*power 3.0.10 software with an alpha value of 0.05 and a power of 90% and based on the study conducted by Arntsen et al. [8] in which the length of maxillary central incisor measurement was 20.86 ± 1.71 and 24.46 ± 1.6 mm in both malocclusion and control groups, respectively. The resulting sample size was a minimum of twelve lateral cephalometric radiographs in each studied category. This number was increased later to a minimum of 14 lateral cephalometric radiographs in each of the included groups.

### Study setting and participants

The sample was collected from out-patients’ clinic records, Hospital of Stomatology, Guangzhou Medical University, China. The records were obtained as pre-operative records for orthodontic patients seeking orthodontic treatment. The subjects of the study were divided into three equal groups based on the vertical facial pattern.

The sample inclusion criteria included several parameters: (1) aged between 18 and 35 years, 2) skeletal Class I (0° < ANB < 5°) [22] with Class I molars relationship,(3) crowding less than 4 mm, and (4) good quality lateral cephalometric radiographs. The exclusion criteria included: (1) bad oral habit (habits like the thumb sucking or tongue thrusting might affect the vertical pattern of growth and the dentoalveolar position of the maxillary anterior segment); (2) congenital missing, supernumerary, fused, or deformed teeth; (3) history of orthodontic treatment; (4) history of maxillofacial trauma; (5) root resorption; (6) history of tooth extraction; (7) systemic diseases or disorders; and (8) deformity of the face or cleft lip and/or palate.

According to the selection criteria, 213 subjects were selected from 9800 fully screened medical and dental records from 2014 to 2019.

### Cephalometric analyses

The lateral cephalometric radiographs were taken using a Soredex (Tuusula, Finland). The lateral cephalometric radiographs were taken with a film-to-focus distance of 180 cm and a film-to-median plane distance of 10 cm. The lateral cephalometric radiographs were traced and measured using CliniView software (version 10.1.2.4, PaloDEx Group Oy, Tuusula, Finland) and standard via a pre-set scale. Mandibular plane angle (SN/MP) was traced using the Sella-Nasion (SN) and Gonion-Menton (MP) lines. The SN/MP was used to classify the subjects into three vertical facial patterns: (1) hyperdivergent (SN/MP > 37°); (2) normodivergent (27° < SN/MP < 37°); and (3) hypodivergent group (SN/MP < 27°) [23].

**Fig. 1:**
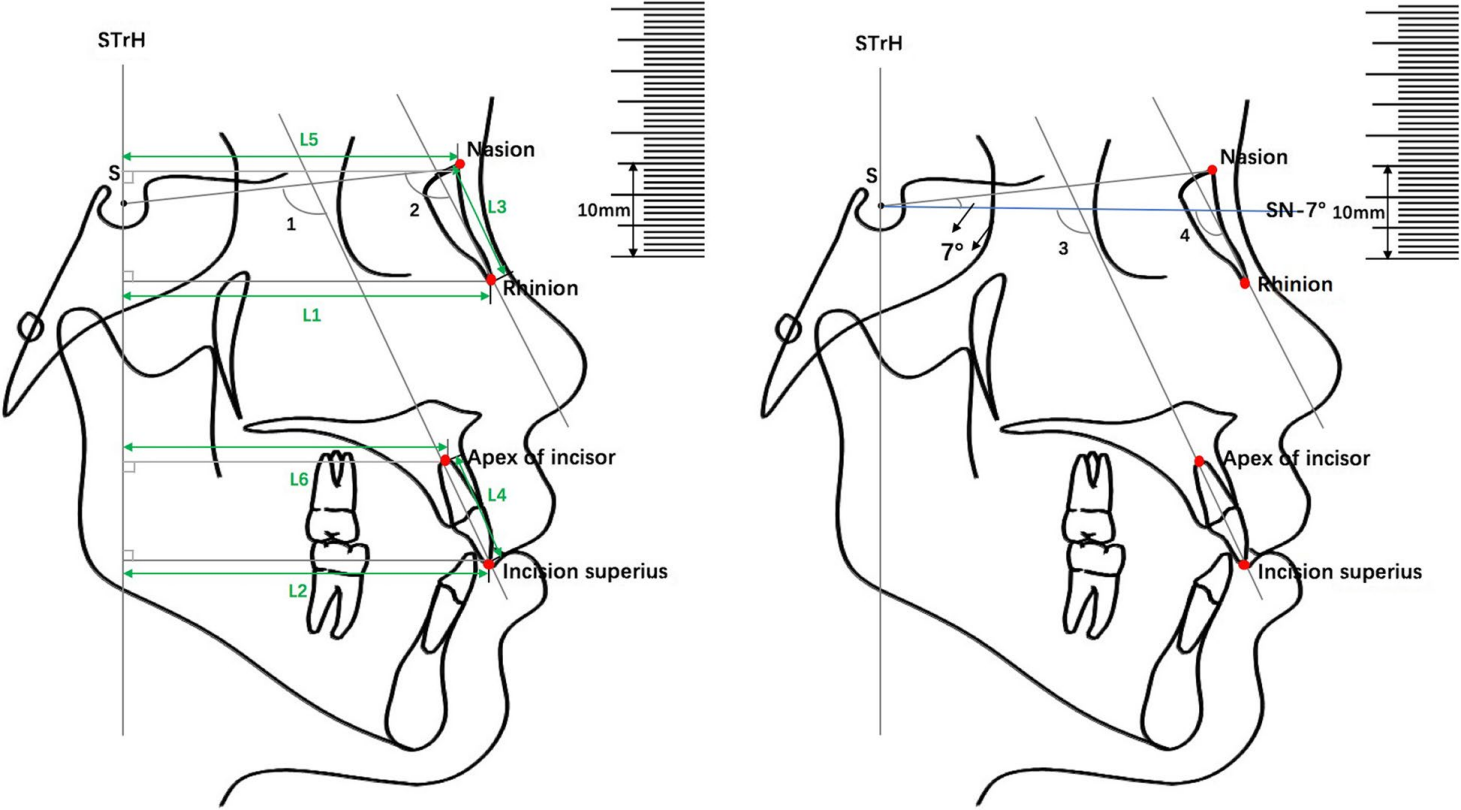
L1: The horizontal distance from tip of the nasal bone to the STrH (N-R-STrH (D)). L2: The horizontal distance from incision superius to the STrH (U1-S-STrH (D)). L3: The length of the nasal bone (N(L)). L4: The length of the maxillary central incisor (U1(L)). L5: The horizontal distance from nasion to the STrH (N–N-STrH (D)). L6: The horizontal distance from apex of maxillary central incisor to the STrH (U1-A-STrH (D)). ∠1: Angle of maxillary central incisor and the SN line. ∠2: Angle of  the nasal bone and the SN line. ∠3: Angle of the maxillary central incisor and the SN-7° line. ∠4: Angle of the nasal bone and the SN-7° line

A plumb line was constructed by hanging weight in front of the electronic screen that displayed on the lateral cephalometric radiographs. A line was traced overlapping the plumb line on the lateral cephalometric radiograph and was defined as a true vertical line (TrH). Another line was drawn through the Sella point parallel to the TrH, which was named the STrH. Craniofacial landmarks were identified on each lateral cephalometry and are presented in Table 1. Reference lines, linear and angular measurements are described in Table 1 and Fig. 1.

**Table 1:** Cephalometric landmarks, measurements and reference lines used in the study

Landmarks/Measurements/ Reference lines	Abbreviation	Descriptions
Sella	S	Centre the sella turcica
Nasion	N	The most receding point of the anterior surface of the frontonasal suture
Rhinion	R	The most prominent and inferior point on the nasal bone
Apex of incisor	Ia	Apex of maxillary central incisor
Incision superius	Is	Incisal edge of the most protruded maxillary central incisor
True vertical reference line	TrH	A line was traced overlapping the plumb line on the lateral cephalometric radiographs
Sella vertical reference line	STrH	A line was drawn through the Sella point parallel to the TrH
Distance from incision superius to STrH	U1-S-STrH (D)	The horizontal distance between the vertical line tangent to the incision superius and the STrH.
Distance from the rhinion to STrH	N-R-STrH (D)	The horizontal distance between the vertical line tangent to the rhinion and the STrH.
Distance from the apex of incisor to STrH	U1-A-STrH (D)	The horizontal distance between the vertical line tangent to the apex of maxillary central incisors and the STrH.
Distance from nasion to STrH	N–N-STrH (D)	The horizontal distance between the vertical line tangent to the nasion and the STrH.
Angle of nasal bone and SN	∠ N-SN	The angle between the axis of the nasal bone (the line from the rhinion point to the midpoint of the base of the nasal bone) and the SN line.
Angle of incisor and SN	∠ U1-SN	The angle between the axis of the maxillary central incisor and the SN line.
SN-7° line	SN-7°	A line drawn with 7° down from the SN line.
Angle of nasal bone and SN-7°	∠ N-SN-7°	The angle between the axis of the nasal bone and the SN-7° line.
Angle of incisor and SN-7°	∠ U1-SN-7°	The angle between the axis of maxillary central incisor and the SN-7°line.
Length of the incisor	U1 (L)	The length from maxillary central incisor, from incisal edge to its apical point of the root.
Length of nasal bone	N (L)	The length from the anterior point of the nasal bone to the midpoint of the base of the nasal bone.
Ratio of angles	A (r)	Angle of nasal bone and SN / Angle of maxillary central incisor and the SN line.
Ratio of angle- SN-7°	A (7°) (r)	Angle of nasal bone and SN-7°/ Angle of maxillary central incisor and the SN-7° line.
Ratio of distance	D (r)	Distance from the tip of the nasal bone to the STrH / Distance from incision superius to the STrH line.
Ratio of length	L (r)	Length of nasal bone / Length of maxillary central incisor.

Based on the measurements of the nasal bone and maxillary central incisors, the ratios were calculated as presented in table 1.

#### Measurement errors

To determine the significant error of the radiographic measurement technique, random lateral cephalometric radiographs of 50 patients were re-measured by another examiner and by the primary investigator three weeks after the first measurements. For each variable, the mean measurement differences between the initial and secondary measurements in addition to the proportion of total variations explained by measurement errors were computed.

### Statistical analysis

All data were collected, tabulated and statistically analyzed using Statistical Package for Social Sciences (SPSS) software, version 21 (IBM Corp., Armonk, NY, USA) for Windows.

Results were presented as mean ± standard deviation (SD). The data was then checked for normal distribution using the Shapiro–Wilk test and homogeneity of the variances.

Pearson’s correlation coefficient was used to assess the correlation between the corresponding variables of the maxillary central incisor and nasal bone in the same vertical facial patterns.

One-way analysis of variance and chi-squared tests were applied to detect significantly different characteristics among the three vertical facial patterns. The SNK-q test was used to compare each pair of the two vertical facial patterns. Reliability analysis was performed using Kappa statistics. The level of significance was set at *P* < 0.05 for all statistical analyses.

## Results

The total number of subjects was 213 subjects (51 Males and 162 Females). The result of intra- and inter-observer reliability showed a very high agreement with an average of 0.085. The general characteristics, such as age and gender distribution, showed no significant differences among the three studied groups. SN/MP was used to classify the vertical facial patterns, and it was significantly different among the three studied groups (Table 2).

**Table 2:** Sample characteristics among the three vertical facial patterns

Facial pattern	SN-MP	Age	Gender		Number (n)
	**(Mean ± SD)**	**(Mean ± SD)**	**Male (%)**	**Female (%)**	
**Hypodivergent**	**25.7 ± 3.61**	**24 ± 4.31**	**17 (23.94%)**	**54 (76.06%)**	**71**
**Normodivergent**	**32.45 ± 1.80**	**24 ± 4.38**	**14 (19.72%)**	**57 (80.28%)**	**71**
**Hyperdivergent**	**38.2 ± 3.40**	**24 ± 4.08**	**20 (28.17%)**	**51 (71.83%)**	**71**
***P-value***	0.000*	**0.416**			

^*^p < 0.05

In terms of the correlation between the nasal bone and maxillary central incisors (Table 3), the distance from the rhinion and maxillary central incisor to the STrH showed a significant positive correlation in the three groups of vertical facial patterns, with *r* values of 0.65 (*p* < 0.001) in the hypodivergent, 0.60 (*p* < 0.001) in the normodivergent, and 0.79 (*p* < 0.05) in the hyperdivergent group.

**Table 3:** Correlation between the nasal bone and maxillary central incisor in the three vertical facial patterns

	**Female**				**Male**			**Total**		
	Measurement	**Mean ± SD**	**R**	***P***	**Mean ± SD**	**R**	***P***	**Mean ± SD**	**R**	***P***
**Hyperdivergent**	**N-R-STrH (D)**	**71.46 ± 3.92**	**0.82**	** > 0.05**	**77.13 ± 1.41**	**0.35**	** < 0.001**	**73.17 ± 4.11**	**0.79**	** < 0.05**
	**U1-S-STrH (D)**	**71.88 ± 5.78**			**73.20 ± 2.27**			**72.60 ± 5.20**		
	**ΔN-SN**	**116.1 ± 5.78**	**0.41**	** < 0.001**	**118.8 ± 1.87**	**0.52**	** < 0.001**	**117.2 ± 5.28**	**0.40**	** < 0.001**
	**ΔU1-SN**	**108.3 ± 5.47**			**123.5 ± 5.98**			**108.5 ± 5.47**		
	**N(L)**	**23.30 ± 2.92**	**0.79**	** < 0.05**	**25.70 ± 1.76**	**0.35**	** < 0.001**	**24.39 ± 2.81**	**0.75**	** < 0.001**
	**U1(L)**	**22.67 ± 1.54**			**22.48 ± 1.57**			**22.65 ± 1.55**		
**Normodivergent**	**N-R-STrH (D)**	**71.63 ± 3.06**	**0.59**	** < 0.05**	**77.51 ± 3.37**	**0.57**	** > 0.05**	**72.48 ± 3.81**	**0.60**	** < 0.01**
	**U1-S-STrH (D)**	**70.30 ± 5.87**			**75.25 ± 5.98**			**71.76 ± 6.27**		
	**ΔN-SN**	**117.2 ± 4.50**	**0.49**	** < 0.001**	**118.1 ± 5.92**	**0.61**	** < 0.001**	**117.8 ± 4.79**	**0.56**	** < 0.001**
	**ΔU1-SN**	**111.1 ± 4.90**			**111.3 ± 8.19**			**111.1 ± 5.71**		
	**N(L)**	**21.59 ± 2.88**	**0.57**	** > 0.05**	**24.23 ± 2.70**	**0.59**	** > 0.05**	**21.90 ± 2.96**	**0.60**	** > 0.05**
	**U1(L)**	**21.98 ± 1.59**			**22.97 ± 2.16**			**22.07 ± 1.81**		
**Hypodivergent**	**N-R-STrH (D)**	**72.38 ± 3.71**	**0.69**	** < 0.01**	**74.85 ± 4.54**	**0.62**	** < 0.05**	**72.91 ± 4.00**	**0.65**	** < 0.01**
	**U1-S-STrH (D)**	**69.70 ± 6.33**			**68.65 ± 5.50**			**69.25 ± 6.07**		
	**ΔN-SN**	**120.7 ± 5.01**	**0.63**	** < 0.001**	**124.3 ± 6.02**	**0.55**	** < 0.001**	**121.75 ± 5.39**	**0.61**	** < 0.001**
	**ΔU1-SN**	**113.9 ± 5.08**			**113.8 ± 6.52**			**113.8 ± 5.48**		
	**N(L)**	**22.34 ± 2.62**	**0.47**	** < 0.05**	**23.54 ± 2.49**	**0.60**	** > 0.05**	**22.62 ± 2.59**	**0.52**	** < 0.01**
	**U1(L)**	**21.49 ± 1.53**			**21.75 ± 1.76**			**21.63 ± 1.59**		

Significance levels: *p* < 0.05

The inclination angle showed a significantly positive correlation between the nasal bone and maxillary central incisor in the three groups, with *r* values 0.61 (*p* < 0.001) in the hypodivergent, 0.56 (*p* < 0.001) in the normodivergent, and 0.40 (*p* < 0.001) in the hyperdivergent group.

The length of the nasal bone and maxillary central incisor was presented a positive correlation in all three groups, with *r* values 0.52 (*p* < 0.01) in the hypodivergent, 0.60 (*p* < 0.01) in the normodivergent, and 0.75 (*p* < 0.001) in the hyperdivergent group.

**Fig. 2:**
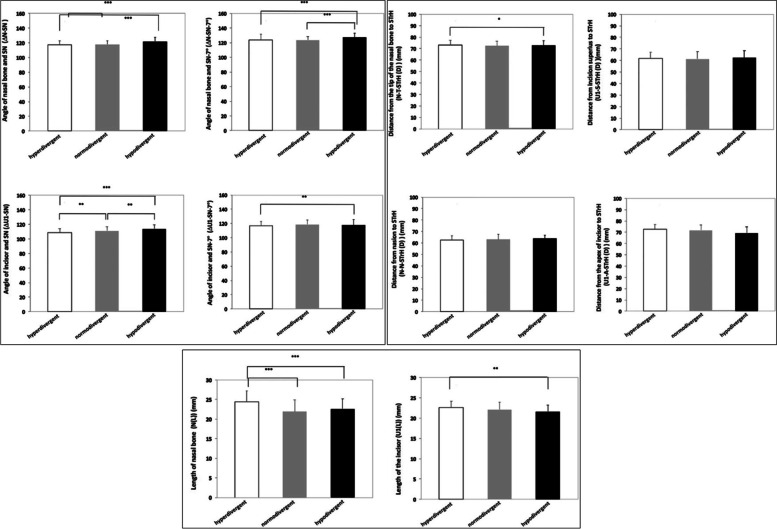
Comparison of the corresponding measurements of the nasal bone and maxillary central incisor among hyperdivergent, normodivergent, and hypodivergent facial pattern (*p < 0.05, **p < 0.01, ***p < 0.001)

The characteristics of the nasal bone and maxillary central incisor were compared in the three vertical facial patterns (Fig. 2).

The inclination angle of the nasal bone in the hyperdivergent and normodivergent groups was significantly smaller than that in the hypodivergent group (*p* < 0.001). The inclination angle of the maxillary central incisor gradually and significantly increased in the hyperdivergent, normodivergent, and hypodivergent groups.

The distance from the rhinion to STrH in the hypodivergent group was significantly shorter than that in the hyperdivergent group (*p* < 0.05). However, the distance from the maxillary central incision superius, nasion, and apex of the maxillary central incisor to the STrH showed no significant differences among the three studied groups. The length of the nasal bone in hyperdivergent was significantly longer than the the other two groups (*p* < 0.001). The length of the maxillary central incisor in the hyperdivergent was significantly longer than that in hypodivergent group (*p* < 0.01).

**Fig. 3:**
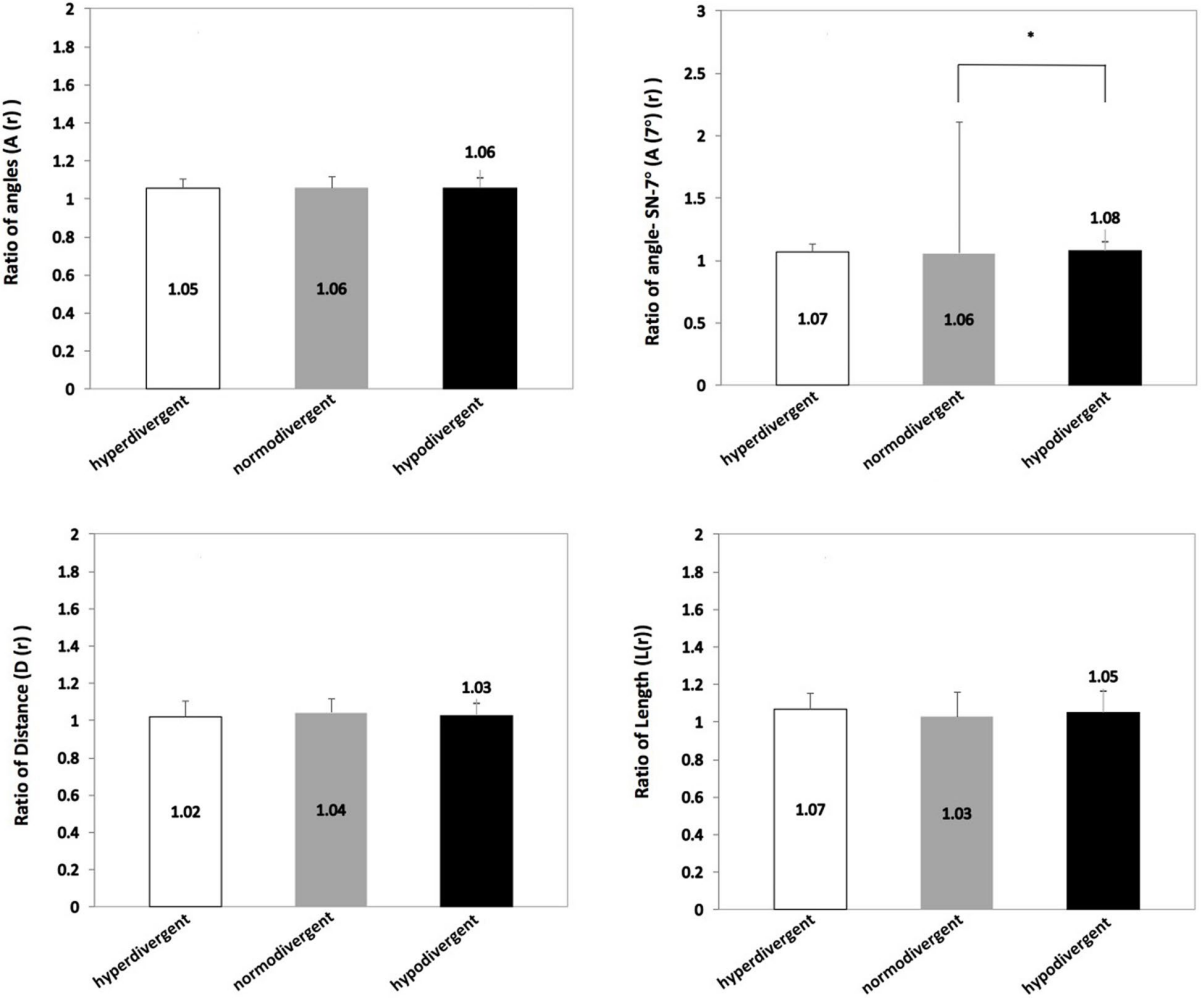
Comparison of the ratio of corresponding measurements of the nasal bone and maxillary central incisor among hyperdivergent, normodivergent, and hypodivergent groups (*p < 0.05)

The ratio of the nasal bone to maxillary central incisor, including the distance to STrH, length, and inclination angle, was not significantly different among the three vertical facial patterns (Fig. 3).

## Discussion

Previous studies have reported that tissues of frontonasal area have a correlation with the maxillary region [8, 24]. For example, Kumaravel et al. [24] measured the horizontal visible iris diameter and width of the maxillary central incisors of an Indian using a standardized image of face and figured out that the eyeballs have a positive correlation with the width of the maxillary central incisors. Also, researchers have focused on the relationship between the nose and maxillary central incisors during craniofacial development. Uppal et al. [25] found malposed incisors in the nose. Additional studies have shown the shape, size, and length of the nose are not only relevant to the maxillary central incisor, but they can also predict the width of these teeth [8, 12]. As the nose and maxillary central incisors are located in the middle of the face [26], they play an important role in facial appearance. Not surprisingly, the relevant measurements in this study also confirmed the correlation between the nasal bone and maxillary central incisors.

In this study, almost all measurement indices of the nose and maxillary central incisors showed a significantly positive correlation. These results implied the similar biological behavior and pattern of the nasal bone and maxillary central incisors during the development of Class I osteofacial type, which is in line with previous studies [9, 11, 12, 27]. Gomes et al. [9] and Sulun et al. [12], for example, selected similar age compositions in their studies to measure the width and shape of the nose. They concluded that the development of the nose and maxillary central incisors is morphologically related. Specifically, the width and shape of the nose could be used to predict and evaluate the shape of these teeth. These previous studies selected the nose as an object instead of selecting the nasal bone, and they measured it on the photos, which is different from the measurement on a standardized X-ray film in this study. Additionally, they all mainly focused on the conventional sagittal measurements rather than inclinations and comprehensive ones [9, 12].

Additionally, the measurements of the nasal bone and maxillary central incisor were compared among different vertical facial patterns in this research. These results showed that the inclination angle of both the nasal bone and maxillary central incisors in the hypodivergent group was larger than that of the other two facial patterns. It can be speculated that hypodivergent patients are more likely to have a higher nose and more prominent anterior teeth. Analogous results were reported by Bou Assi et al. [28], who pointed out that the maxillary incisors show compensative labial inclination more significantly in the hypodivergent versus the hyperdivergent group. Moreover, the distances from the nasal root point and the apex of maxillary central incisors to the true vertical line were compared among different vertical facial patterns, and they showed no significant difference. The distance may be related to the overall sagittal diameter of craniocerebrum and craniofacial instead of the sagittal development of the nasal bone and maxillary central incisors [29].

Nehra et al. [29] have investigated the relationship between nasal morphology and vertical maxillary skeletal pattern, and it was concluded that the nasal length is positively correlated with anterior and posterior facial height. Intriguingly, the results of this study showed that the length of both the nasal bone and maxillary central incisors in hyperdivergent group was significantly higher than that in the normodivergent and hypodivergent groups. This finding indicates that hyperdivergent patients are more likely to have a longer nose and maxillary central incisors; in contrast, the nose and maxillary central incisors in hypodivergent patients are relatively shorter. This difference may be related to the vertical growth of the face. These features may well match the facial pattern to form a harmonious face [26], but the relationship and principle are worth exploring further.

In this study, the corresponding measurement values of the nasal bone and maxillary central incisors were calculated as proportions. Each ratio was compared in  the three different vertical facial patterns. No statistical differences among each group were found. A comprehensive analysis of the previous results of this study concluded that the growth direction and length of the nasal bone and maxillary central incisors in Class I subjects were closely related, which was irrelevant to the vertical growth of the face. Embryonic origins may mainly account for the characteristics and behavior of facial growth and development.

The growth patterns and characteristics of the craniofacial morphology are constructed naturally. Growth and development compensate and coordinate the facial features with growth patterns, which make the face more harmonious and natural. Therefore, understanding of characteristics and the relationship of facial structure with its growth patterns can provide references for the restoration of missing anterior teeth and reconstruction of the shape and height of a defective nose [30]. More and more people pursue taller noses and more upright maxillary central incisors. However, these features should be designed according to the overall growth characteristics of face in addition to comprehensive coordination of bone and facial features.

The limitation of this study was the lopsided gender ratio, which consisted of less male subjects. This difference might have been due to the larger number of female orthodontic patients who generally number more than male patients. The influence of gender on the nose and incisors remains unclear. However, from the perspective of growth and development and facial structure characteristics [31, 32], gender is bound to have an influence on the nose and incisors. Another limitation is the use of two dimensional imaging method to evaluate a three dimensional object, so it is recommended to consider the effect of age and the use of more advanced modality in any future research.

## Conclusion

A positive correlation between the nasal bone and maxillary central incisors on the length and inclination in Class I patients was found. This correlation is irrelevant to vertical facial patterns. The development of the nasal bone and maxillary central incisors are shorter and more inclined in hypodivergent patients in contrast to hyperdivergent patients. The clinical implication included the possible use of the maxillary central incisors and the nasal bone interchangeably as a clinical guide during dental restoration or nasal reconstruction, respectively.

## Data Availability

All data are available in the manuscript.
